# IgG positive food elimination diet altered gut microbiota, decreased zonulin in migraine adults: a pilot dietary intervention trial

**DOI:** 10.3389/fnut.2026.1878772

**Published:** 2026-08-12

**Authors:** Zhi-Ming Zhao, Fu-Jun Wan, Mei-Mei Yang, Bao-Li Ning, Tao Song, Jun Fu

**Affiliations:** 1 Health Center of Screening and Prevention of Diseases, The First Affiliated Hospital of Harbin Medical University, Harbin, Heilongjiang, China; 2 Department of Neurology, The Fourth Affiliated Hospital of Harbin Medical University, Harbin, Heilongjiang, China

**Keywords:** Bifidobacterium, Firmicutes, gut microbiota, microbiota gut brain axis, migraine

## Abstract

**Background and aims:**

Gut microbiota and intestinal permeability may play a role in food specific IgG antibodies mediated delayed hypersensitivity in migraine patients. We aimed to evaluate the effect of food specific IgG-guided elimination strategy by assessing the changes in gut microbiota, zonulin as well as their correlation with symptoms, IL-6, TNF-α and CGRP.

**Methods:**

The study included 52 migraine patients in true diet group (excluding IgG positive foods) and 46 controls in sham diet group (excluding alternative IgG negative foods). The gut microbiota were evaluated by the 16S rRNA amplicon sequencing. Relative abundance analyses, α-diversity and β-diversity were implemented using QIIME 2. Zonulin, IL-6, TNF-α and CGRP was assessed using ELISA. Questionnaire surveys were used to investigate symptoms of migraine and its comorbidities.

**Results:**

At the end of 12 weeks, there were significant differences in β-diversity and the Simpson index (α-diversity) of gut microbiota in the true diet group. The relative abundance of Bacteroidetes decreased while that of Firmicutes as well as Bifidobacterium and Butyricicoccus increased in the true diet group. The true diet group had a larger reduction in zonulin compared to the sham diet group. The changes after the true diet of the relative abundance of certain gut microbiota were significantly correlated with MSQ, PSQI, IL-6, CGRP and zonulin.

**Conclusion:**

IgG positive food elimination diet altered gut microbiota, which was associated with decreased zonulin, CGRP and IL-6, and improvements in migraine and sleep.

**Clinical trial registration:**

Chinese Clinical Trial Registry (Chictr.org.cn, identifier ChiCTR2000039278).

## 1 Introduction

Migraine and its comorbidities such as depression, anxiety, sleep disorders, and gastrointe can cause severe disability in patients (1, 2). Our Prior studies have have identified that food specific IgG antibodies mediated delayed hypersensitivity is associated with migraine and its comorbidities (3, 4). IgG positive food elimination diet was beneficial

to migraine and its comorbidities and reduced IL-6, TNF-α, and CGRP, which might be associated with the alleviated systemic chronic inflammation and downregulation of the sensitivity of trigeminal nerve endings (5).

Recent discoveries further highlight the gut microbiota (i.e., the biological diversity, abundance, or combinations of both components) changes in migraine and its comorbidities (6–9). Imbalanced gut microbiome seems to be shared mechanism or risk factor of migraine and its comorbidities (10). The types of microbiota colonized in the intestine play a role in regulating immunity, which may be related to the pathogenesis of migraine (11, 12). The gut microbiota influence inflammation, neurotransmitter production, and neuronal excitability as an important modulator of migraine susceptibility via the gut–brain axis (13).

In addition, Gut microbiota dysbiosis may lead to the release of zonulin, a human protein involved in the integrity and functioning of both intestinal-epithelial barrier and blood-brain barrier (BBB) by regulating tight junction molecular assembly (14).

By this background, we compared the effects of two food specific IgG-guided elimination strategy (fasting IgG positive food or sham diet) on gut microbiota and zonulin of migraine adults by a sham-controlled randomized study. Then we further explored the correlation of gut microbiota with clinical symptoms and serum biomarkers as exploratory findings.

## 2 Material and methods

### 2.1 Study design and dietary intervention

This was a single blinded sham-controlled randomized trial. The patients were randomly assigned to the “true” dietary group (eliminating IgG positive foods) or “sham” dietary group (eliminating IgG negative foods). More inclusion criteria and intervention specific details of this cohort of patients were reported previously (5).

As previously described (5), seven questionnaires were administered to measure the migraine-related disability, impact on quality of life, severity, and the comorbidities at baseline and the end of 12 weeks: (1) Migraine Disability Assessment (MIDAS) and Visual Analog Scale (VAS) (2) Headache Impact Test-6 (HIT-6) (3) Migraine-Specific Quality of Life Questionnaire version 2.1 (MSQ), by raw dimension scores (4) Self-Rating Anxiety Scale (SAS) (5) Self-Rating Depression Scale (SDS) (6) Pittsburgh Sleep Quality Index (PSQI) (7) Gastrointestinal Symptom Rating Scale (GSRS).

### 2.2 Blood biomarkers detection

Blood samples were collected at baseline and the 12-week endpoint. We used enzyme-linked immunosorbent assays (ELISA) to measure the serum concentration of 14 different types of food-specific IgG antibodies (Bioeurope GmbH, Germany), IL-6, TNF-α, CGRP, 5-HT (BIM, San Francisco, CA) and IL-10, VIP, zonulin (ShangHai HengYuan Biological Technology Co.,Ltd). All the ELISA were performed according to the manufacturer's recommendations using Biochrom Anthos 2010 Microplate Reader.

### 2.3 Gut microbiota analysis

Random fecal samples were collected at baseline and the 12-week endpoint. DNA extraction was performed using the TIANamp Stool DNA Kit (TIANGEN Biotech, Beijing, China) according to manufacturer's instructions. Then study changes in gut microbiota by 16S ribosomal RNA amplicon sequencing. After PCR amplification, sample mixing and library construction, the qualified libraries will be sequenced using Illumina Miseq high-throughput sequencing platform, and the raw data obtained from sequencing will be used for later information analysis.

To begin with, the raw data was filtered by removing the unqualified reads from the raw reads. The data was divided into different sample data based on the Barcode sequence, and the PCR amplification primer sequence and Adaptor contaminated data were truncated. After quality control, the clean reads were obtained. We removed low-quality sequences and chimeras from the split Pair End Reads data using QIIME2 DADA2.0 to generate an OTU table. OTU length filtering was performed to remove sequences with OTU lengths less than 300bp obtained after truncation (15). Afterwards, based on valid data, OTUs clustering and species classification annotation were performed. The ucluster algorithm was used to compare and annotate OTUs with the Greengenes database. Then we combined OTUs with species annotation to obtain the OTUs data contained in each sample. Simultaneously, the OTUs were aligned assigned taxonomy at the phylum, class, order, family, and genus levels. Finally, diversity indices and relative abundance analysis were conducted.

### 2.4 Statistical analysis

Data were analyzed using SAS 9.4 (SAS Institute, Inc., Cary, NC, USA). A significance level of 0.05 was used. Paired t tests were used to assess the change of relative abundance of gut microbiota, serum concentrations of zonluin over time within each diet group; two sample independent t tests were used to compare these changes between two diet groups.

The bioinformatics analysis software QIIME (Quantitative Insights Into Microbial Ecology) was used to analyze the relative abundance, alpha diversity, beta diversity, and PCoA (Principal co-ordinates analysis) of gut microbiota. Spearman correlation analysis was used to investigate the correlation of gut microbiota with clinical symptoms and serum biomarkers changes before and after dietary intervention. Correlation analysis was visualized using heatmap. Only the microbiota that were significantly different in the related group comparisons were used, to limit the size of the graph and to filter the output information.

## 3 Results

### 3.1 Patients

**Figure 1:**
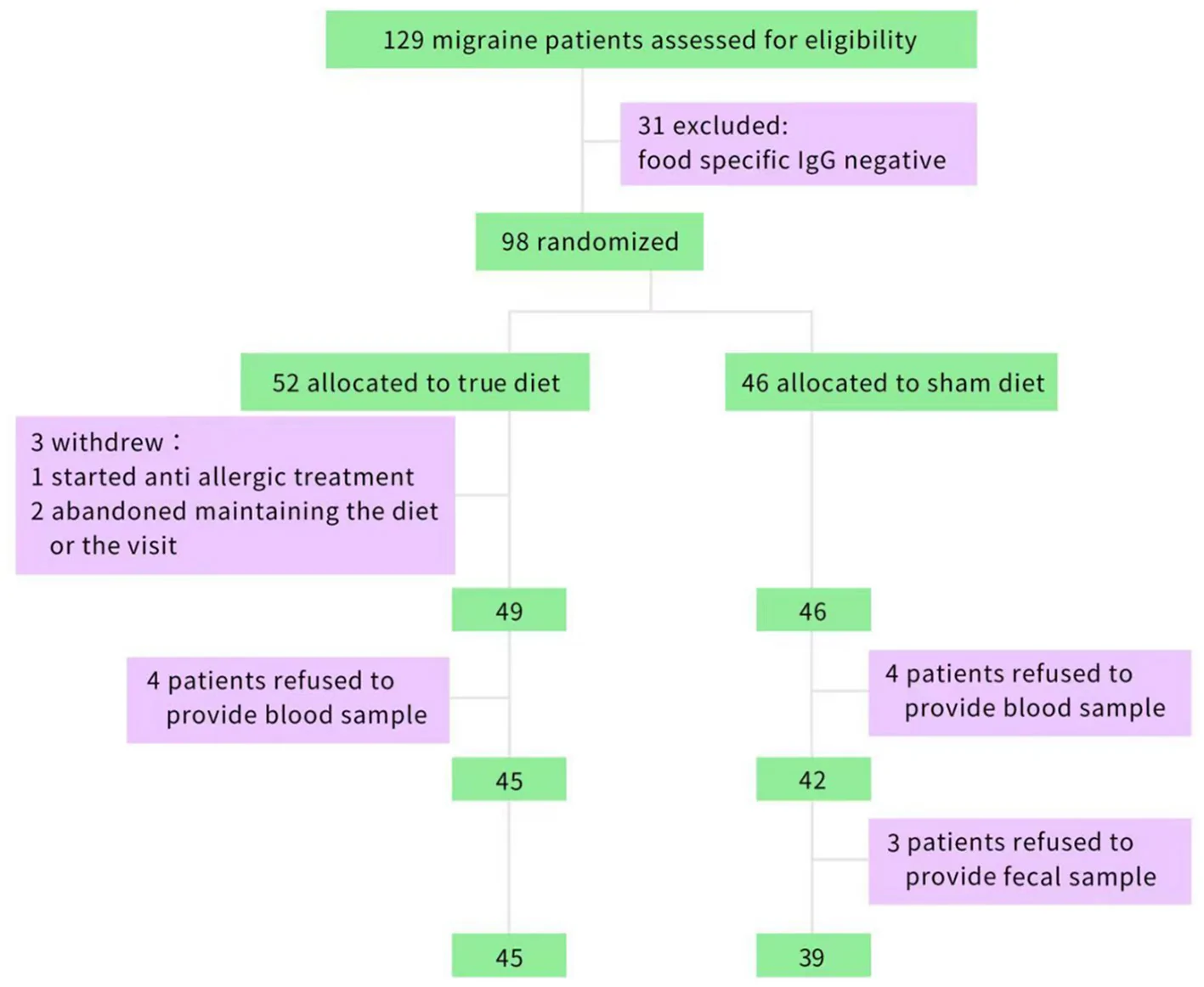
Study flow diagram.

From October 28^th^, 2020 to March 11^th^, 2021, 129 migraine patients were consecutively recruited at the Department of Health Center of Screening and Prevention of Diseases, the First Affiliated Hospital of Harbin Medical University. After the first 14 food specific IgG antibodies test, 98 patients had at least one IgG positive, among whom 52 were randomly assigned to the true diet group, and 46 to the sham diet group. At the end of 12 weeks, 84 patients completed post-intervention questionnaires and provided blood and focal samples (Figure 1). There was no significant difference in characteristics of patients between the two groups at baseline (Supplementary Table 1).

### 3.2 Comparison of zonulin changes between the two diets

After 12 weeks, in the true diet group, zonulin levels decreased from 399.5 (STD: 364.82) pg/ml to 253.3 (STD: 244.67) pg/ml, with a mean change of −146.1 pg/ml (95% CI: −257.2, −35.1), and a statistically significant change was detected (*P* = 0.0111). There was no statistically significant change in the sham diet group (*P* = 0.0977). The comparison of zonulin changes between the two groups showed a significant difference (*P* = 0.0025).

The concentrations comparison of CGRP, TNF-α, and IL-6 of this cohort were reported previously (5).

### 3.3 Comparison of two diets on gut microbiota

#### 3.3.1 Diversity analysis

**Figure 2:**
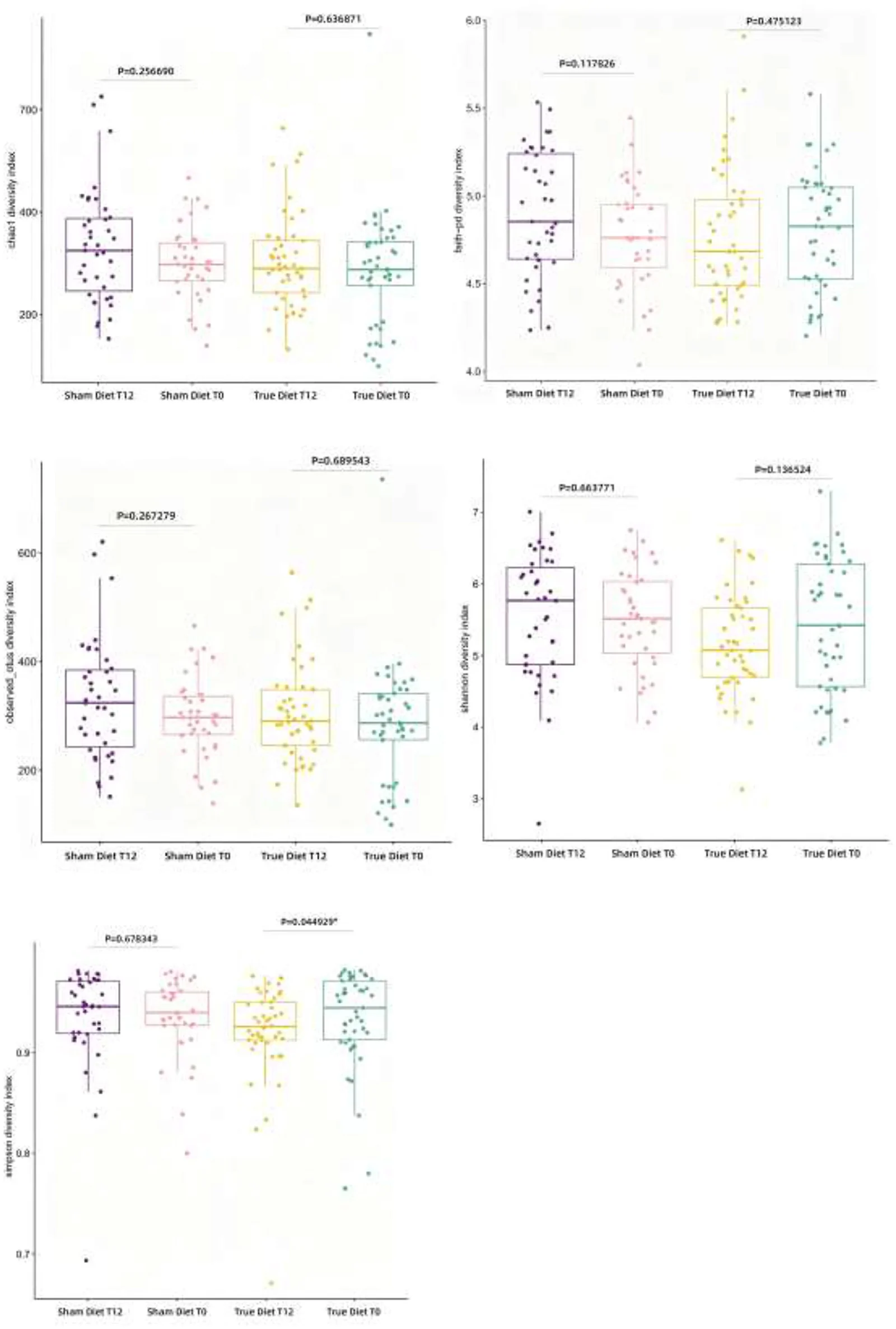
Box plots of alpha diversity indices in two groups. Each box from bottom to top indicates the Minimum, Lower quartile, Median, Upper quartile and Maximum.

Only the Simpson index significantly changed (H = 4.021311, *P* = 0.044929) in the true diet group after 12 weeks. As the other four alpha diversity indices, including Chao, Faith_PD, observed_OTUs and Shannon, there was no significant difference (P>0.05) between before and after the two diets (Figure 2 and Supplementary Table 2).

The change in beta diversity of gut microbiota after 12 weeks of intervention was measured using Bray Curtis distance, weighted UniFrac distance and unweighted UniFrac distance analysis (Supplementary Table 3). The results showed that there was a significant difference in these 3 indices between baseline and endpoint in the true diet group(*p* values were 0.046, 0.020, and 0.001).

**Figure 3:**
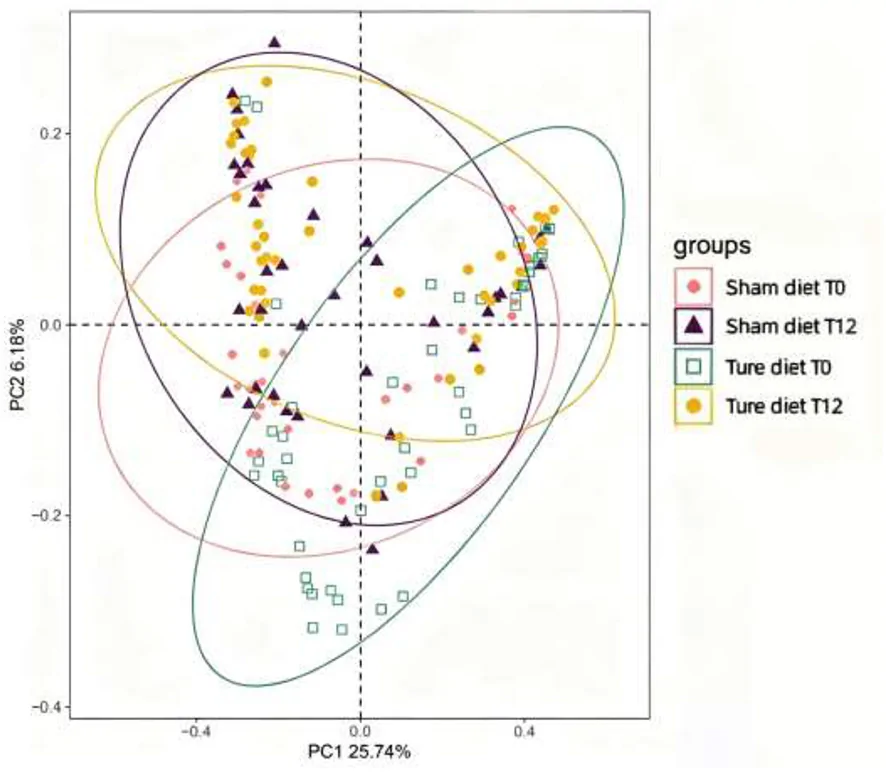
Cluster analysis by PCoA. The axes represent the two dimensions explaining the greatest proportion of variance in the communities. Each symbol represents a sample, and the points of different colors or shapes in the figure represent different groups. The scales on the horizontal and vertical axes are relative distances.

PCoA clustering analysis showed changes in the composition of gut microbiota in the true diet group (Figure 3). There was a significant intra group separation and more dispersed sample distribution before and after true diet. The similarity in microbial structure and composition within the true diet group before and after true diet was lower than that in the control group, indicating that true diet can increase the differences in gut microbiota among individuals to a certain extent.

#### 3.3.2 Taxa alteration between two groups

**Figure 4:**
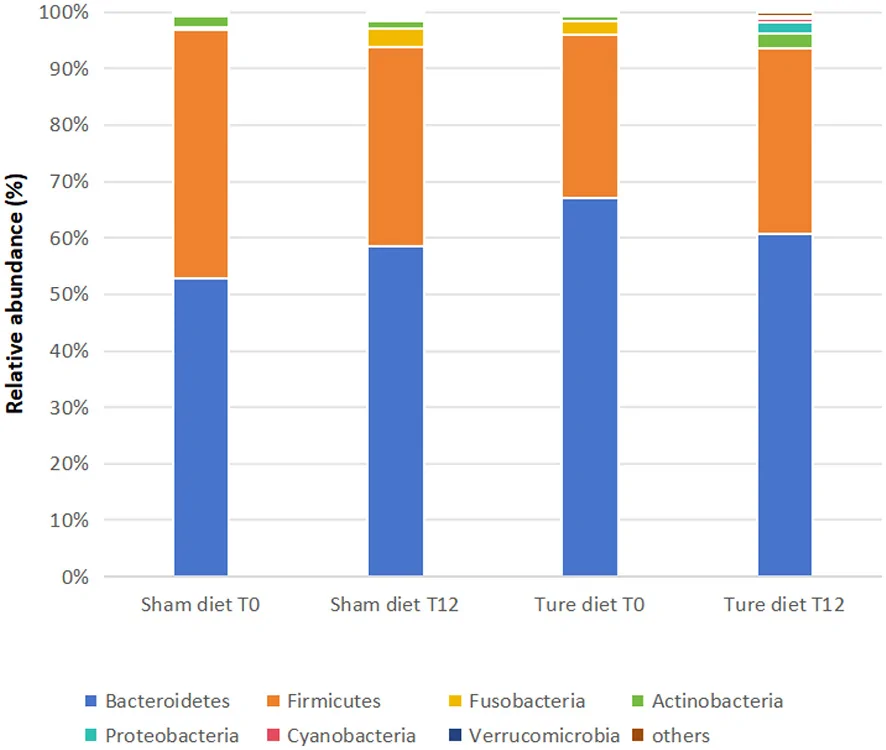
Distribution of bacterial community structure at the phylum level.

The analysis results at the phylum, class, order, family and genus levels between two groups are summarized in Table 1. The top seven phylum with the highest relative abundance were Bacteroidetes, Firmicutes, Fusobacteria, Actinobacteria, Proteobacteria, Cyanobacteria, and Verrucomicobia. Despite there were some changes before and after two diets, Bacteroidetes and Firmicutes were always the two phylum with the highest relative abundance, accounting for over 90% of the total sequence. However, as shown in Figure 4, the two groups showed different trends of increase/decrease before and after dietary intervention: the Bacteroidetes decreased while the Firmicutes increased after a true diet, but after a sham diet, the Firmicutes decreased while the Bacteroidetes increased (*p* values were 0.0399 and 0.0145, respectively). In addition, the true diet group showed a significant increase in the Actinobacteria (*p* = 0.0037).

**Table 1:** Relative abundance of gut microbiota of two groups (%).

Taxonomic Classification	Sham diet (*N* = 39)				True diet (*N* = 45)				*P* value
	Baseline	Week 12	Change (95% CI)	*P* value [1]	Baseline	Week 12	Change (95% CI)	*P* value [1]	[2]
Phylum									
Bacteroidetes	52.91 (23.390)	58.47 (18.029)	5.56 (−3.00, 14.12)	0.1962	67.03 (15.626)	60.77 (22.626)	−6.26 (−13.85, 1.33)	0.1034	0.0399
Firmicutes	43.97 (22.450)	35.23 (17.041)	−8.74(−17.31, −0.17)	0.0235	28.92 (13.51)	32.82 (20.749)	3.91(−2.56, 10.37)	0.3371	0.0145
Fusobacteria	0.40 (1.721)	3.32 (8.828)	2.92(0.30, 5.52)	0.0296	2.51 (8.336)	2.68 (8.397)	0.17 (−3.46, 3.78)	0.9282	0.2170
Actinobacteria	1.96 (3.813)	1.37 (2.113)	−0.58 (−1.62, 0.45)	0.2596	0.73 (1.794)	1.84 (2.339)	1.11 (0.65, 1.58)	< 0.0001	0.0037
Proteobacteria	0.26 (0.659)	0.40 (0.550)	0.14 (−0.07, 0.35)	0.1821	0.30 (0.777)	0.78 (1.409)	0.48(0.05, 0.91)	0.0305	0.1628
Cyanobacteria	0.17 (0.861)	0.36 (1.976)	0.19 (−0.52, 0.89)	0.5983	0.38 (1.953)	0.39 (1.677)	0.01(−0.78, 0.79)	0.9846	0.7345
Verrucomicrobia	0.22 (1.172)	0.37 (1.246)	0.15 (−0.42, 0.72)	0.5975	0.01 (0.034)	0.37 (1.240)	0.36(−0.02, 0.73)	0.0607	0.5418
Class									
Bacteroidia	52.90 (23.396)	58.45 (18.029)	5.55(−3.02, 14.11)	0.1976	67.02 (15.624)	60.73 (22.628)	−6.30 (−13.88, 1.29)	0.1015	0.0397
Clostridia	42.88 (22.109)	34.25 (16.686)	−8.63 (−17.01, −0.25)	0.0438	28.52 (13.265)	31.16 (19.197)	2.64 (−3.40, 8.67)	0.3831	0.0306
Fusobacteriia	0.40 (1.721)	3.32 (8.828)	2.91(0.30, 5.52)	0.0296	2.51 (8.336)	2.68 (8.397)	0.16 (−3.46, 3.78)	0.9282	0.2170
Actinobacteria	1.74 (3.703)	1.07 (1.999)	−0.67(−1.67, 0.34)	0.1855	0.60 (1.743)	1.36 (2.019)	0.76 (0.46, 1.06)	< 0.0001	0.0084
Erysipelotrichi	1.04 (1.950)	0.91 (1.524)	−0.12 (−0.72, 0.47)	0.6790	0.37 (0.591)	1.51 (3.433)	1.14 (0.09, 2.20)	0.0343	0.0387
Alphaproteobacteria	0.23 (0.623)	0.30 (0.430)	−0.08 (−0.13, 0.28)	0.4492	0.27 (0.730)	0.67 (1.328)	0.41 (−0.02, 0.83)	0.0584	0.1579
Coriobacteriia	0.21 (0.311)	0.27 (0.493)	0.057 (−0.13, 0.25)	0.5515	0.11 (0.153)	0.45 (1.146)	0.33 (−0.01, 0.68)	0.0583	0.1628
4C0d−2	0.15 (0.863)	0.34 (1.977)	0.20 (−0.51, 0.90)	05733	0.00 (0.00)	0.37 (1.240)	0.35(−0.15, 0.86)	0.1644	0.7166
Verrucomicrobiae	0.22 (1.172)	0.37 (1.247)	0.14(−0.43, 0.71)	0.6101	0.01 (0.033)	0.37 (1.240)	0.36(−0.02, 0.73)	0.0605	0.5314
Order									
Bacteroidales	52.90 (23.396)	58.45 (18.029)	5.55(−3.02, 14.11)	0.1976	67.02 (15.624)	60.73 (22.628)	−6.30 (−13.88, 1.29)	0.1015	0.0397
Clostridiales	42.88 (22.109)	34.25 (16.684)	−8.64 (−17.01, −0.26)	0.0437	28.52 (13.265)	31.13 (19.227)	2.61 (−3.43, 8.64)	0.3884	0.0188
Fusobacteriales	0.40 (1.721)	3.32 (8.828)	2.91(0.30, 5.52)	0.0296	2.51 (8.336)	2.68 (8.397)	0.16 (−3.46, 3.78)	0.9282	0.2170
Bifidobacteriales	1.66 (3.712)	0.94 (2.008)	−0.73 (−1.72, 0.28)	0.1510	0.53 (1.700)	1.09 (2.031)	0.57 (0.29, 0.85)	0.0002	0.0156
Erysipelotrichales	1.04 (1.950)	0.91 (1.524)	−0.12 (−0.72, −0.47)	0.6790	0.37 (0.591)	1.51 (3.433)	1.14 (0.09, 2.20)	0.0343	0.0387
Family									
Prevotellaceae	25.39 (30.188)	25.66 (28.069)	0.27 (−9.30, 9.85)	0.9541	48.47 (25.766)	33.05 (34.565)	−15.42 (−24.55, −6.30)	0.0014	0.0188
Bacteroidaceae	22.89 (21.158)	26.58 (20.582)	3.69 (−5.40, 12.78)	0.4165	15.63 (13.341)	22.35 (21.390)	6.72 (0.10, 13.34)	0.0467	0.5878
Lachnospiraceae	25.83 (18.711)	16.18 (9.955)	−9.65 (−16.81, −2.48)	0.096	14.60 (6.896)	16.521 (11.306)	1.92 (−1.73, 5.57)	0.2945	0.0051
Ruminococcaceae	13.63 (11.418)	13.35 (10.407)	−0.28 (−5.12, 4.56)	0.9067	10.63 (8.133)	10.18 (10.123)	−0.45 (−4.16, 3.26)	0.8075	0.9555
Genus									
Prevotella	25.38 (30.184)	25.65 (28.071)	0.27 (−9.31, 9.85)	0.9552	48.46 (25.764)	33.03 (34.549)	−15.43 (−24.55, −6.30)	0.0014	0.0188
Bacteroides	22.89 (21.158)	26.58 (20.582)	3.69 (−5.40, 12.78)	0.4165	15.63 (13.342)	22.35 (21.390)	6.72 (0.10, 13.33)	0.0468	0.5879
Faecalibacterium	7.07 (7.344)	6.47 (5.345)	−0.60 (−3.30, 2.10)	0.6553	7.16 (6.849)	4.98 (8.280)	−2.18 (−5.25, 0.90)	0.1602	0.4382
Roseburia	6.31 (5.464)	5.45 (3.800)	−0.86 (−3.23, 1.51)	0.4670	4.95 (2.861)	5.40 (4.512)	0.45 (−1.04, 1.95)	0.5442	0.3466
Ruminococcus	7.84 (14.668)	2.22 (1.819)	−5.62 (−10.49, −0.75)	0.0249	1.62 (1.281)	4.44 (7.878)	2.82 (0.44, 5.21)	0.0216	0.0026
Clostridium	4.53 (3.062)	3.77 (3.274)	−0.76 (−2.32, 0.80)	0.3308	3.06 (1.340)	3.33 (2.928)	0.26 (−0.69, 1.21)	0.5820	0.2628
Alistipes	1.82 (2.918)	1.69 (2.723)	−0.13 (−1.38, 1.12)	0.8331	0.48 (0.627)	1.56 (2.444)	1.08 (0.34, 1.81)	0.0050	0.0968
Bifidobacterium	1.66 (3.712)	0.94 (2.009)	−0.72 (−1.72, 0.27)	0.1502	0.53 (1.700)	1.08 (2.033)	0.56 (0.28, 0.84)	0.0002	0.0161
Dorea	1.51 (2.630)	0.43 (0.267)	−1.07 (−1.94, −0.21)	0.0167	0.62 (0.852)	0.67 (0.990)	0.05 (−0.35, 0.45)	0.8054	0.0211
Macellibacteroides	0.53 (0.704)	1.12 (1.517)	0.59 (0.08, 1.10)	0.0241	0.38 (0.365)	0.68 (0.825)	0.31 (0.07, 0.55)	0.0113	0.3123
Gemmiger	0.38 (0.276)	0.32 (0.341)	0.02 (−0.15, 0.12)	0.8124	0.28 (0.189)	0.91 (2.879)	0.63 (−0.23, 1.49)	0.1472	0.1418
Coprococcus	0.31 (0.240)	0.28 (0.371)	−0.03(−0.18, 0.12)	0.6847	0.23 (0.224)	0.29 (0.454)	0.06 (−0.07, 0.20)	0.3383	0.3445
Collinsella	0.16 (0.304)	0.22 (0.451)	0.06(−0.13, 0.25)	0.5123	0.10 (0.141)	0.27 (0.594)	0.17 (−0.01, 0.36)	0.0636	0.3887
Barnesiella	0.12 (0.322)	0.20 (0.457)	0.08 (−0.10, 0.27)	0.3700	0.18 (0.375)	0.18 (0.302)	−0.01 (−0.15, 0.14)	0.9372	0.4488
Butyricicoccus	0.18 (0.197)	0.16 (0.100)	−0.02 (−0.09, 0.04)	0.4643	0.10 (0.079)	0.22 (0.258)	0.12 (0.04, 0.20)	0.0055	0.0072
Paraprevotella	0.16 (0.561)	0.11 (0.115)	−0.05 (−0.24, 0.14)	0.6281	0.11 (0.100)	0.20 (0.549)	0.09 (−0.08, 0.25)	0.2874	0.2846
Lactonifactor	0.32 (0.583)	0.06 (0.072)	−0.26 (−0.44, −0.07)	0.0077	0.10 (0.096)	0.10 (0.181)	0.00 (−0.06, 0.05)	0.8852	0.0110
Butyricimonas	0.08 (0.122)	0.11 (0.139)	0.03 (−0.02, 0.08)	0.1877	0.13 (0.329)	0.05 (0.101)	−0.08 (−0.18, 0.02)	0.1171	0.0464
Akkermansia	0.22 (1.172)	0.37 (1.247)	0.14 (−0.43, 0.71)	0.6101	0.01 (0.033)	0.37 (1.240)	0.36 (−0.02, 0.73)	0.0605	0.5314

[1] *p* values were reported for the paired *t* test used to compare the baseline and 12-weeks follow up measures. [2] *p* values were reported for the two independent sample *t* test used to compare the two groups in the change between baseline and 12-weeks follow up.

At the class and order levels, the dominant gut microbiota before and after dietary intervention were Bacteroides_Bacteroidia and Clostridia_Clostridiales. Compared with baseline, after 12 weeks of dietary intervention, the true diet group showed a decrease in Bacteroides_Bacteroidia but a significant increase in Actinobacteria_Bifidobacteriales and Erysipelotrichi_Erysipelotrichales, which was significantly different from the sham diet group. The Clostridia_Clostridiales in the sham diet group significantly reduced, which was significantly different from the true diet group.

At the family level, compared with baseline, after 12 weeks, the true diet group showed a significant decrease in Prevotellaceae (*P* = 0.0014) and an increase in Molluscaceae, with a significant difference between two groups (*P* = 0188, *P* = 0.0051).

**Figure 5:**
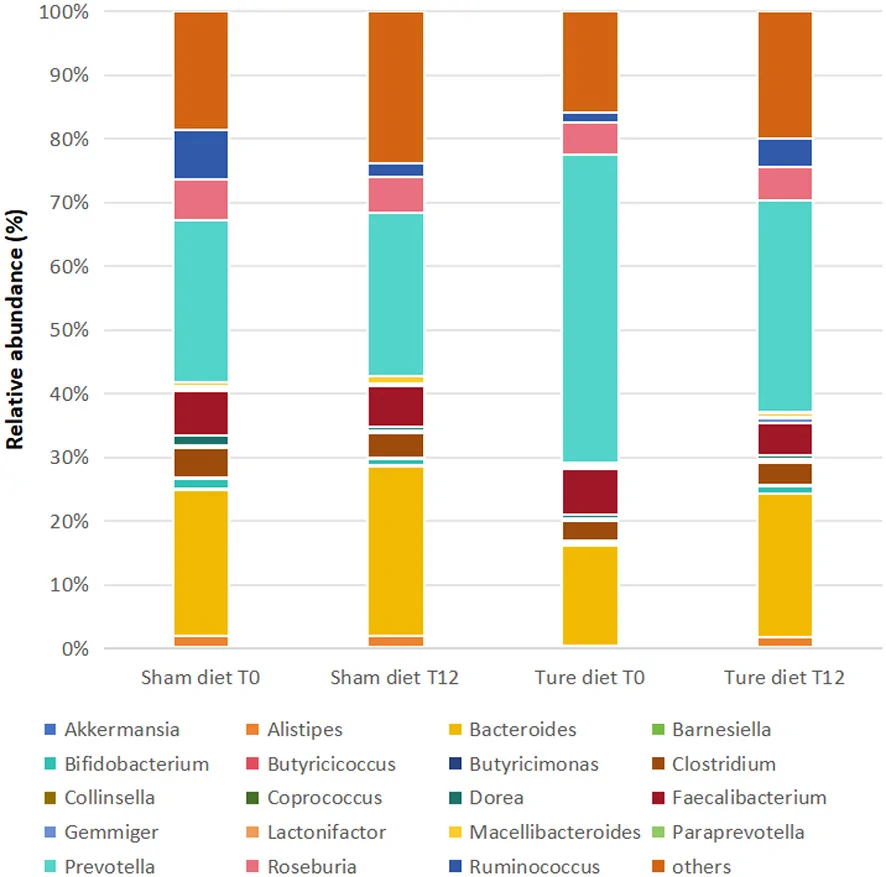
Distribution of bacterial community structure at the level of genus.

At the genus level, after 12 weeks of dietary intervention, the true diet group showed a significant decrease in Prevotella was significantly reduced compared with baseline (*P* = 0.0014), while the Ruminococcus, Bifidobacterium, and Butyricicoccus were significantly increased (*P* = 0.0216, *P* = 0.0002, *P* = 0.0055), with significant differences compared to the sham diet group (*P* = 0.0188, *P* = 0.0026, *P* = 0.0161, *P* = 0.0072); The sham diet group showed significant reductions in Ruminococcus, Dorea, and Lactonifactor (*P* = 0.0249, *P* = 0.0167, *P* = 0.0077), with significant differences compared to the true diet group (*P* = 0.0026, *P* = 0.0281, *P* = 0.0110) (Figure 5).

#### 3.3.3 Correlation between gut microbiota and clinical symptoms, serum biomarkers

**Figure 6:**
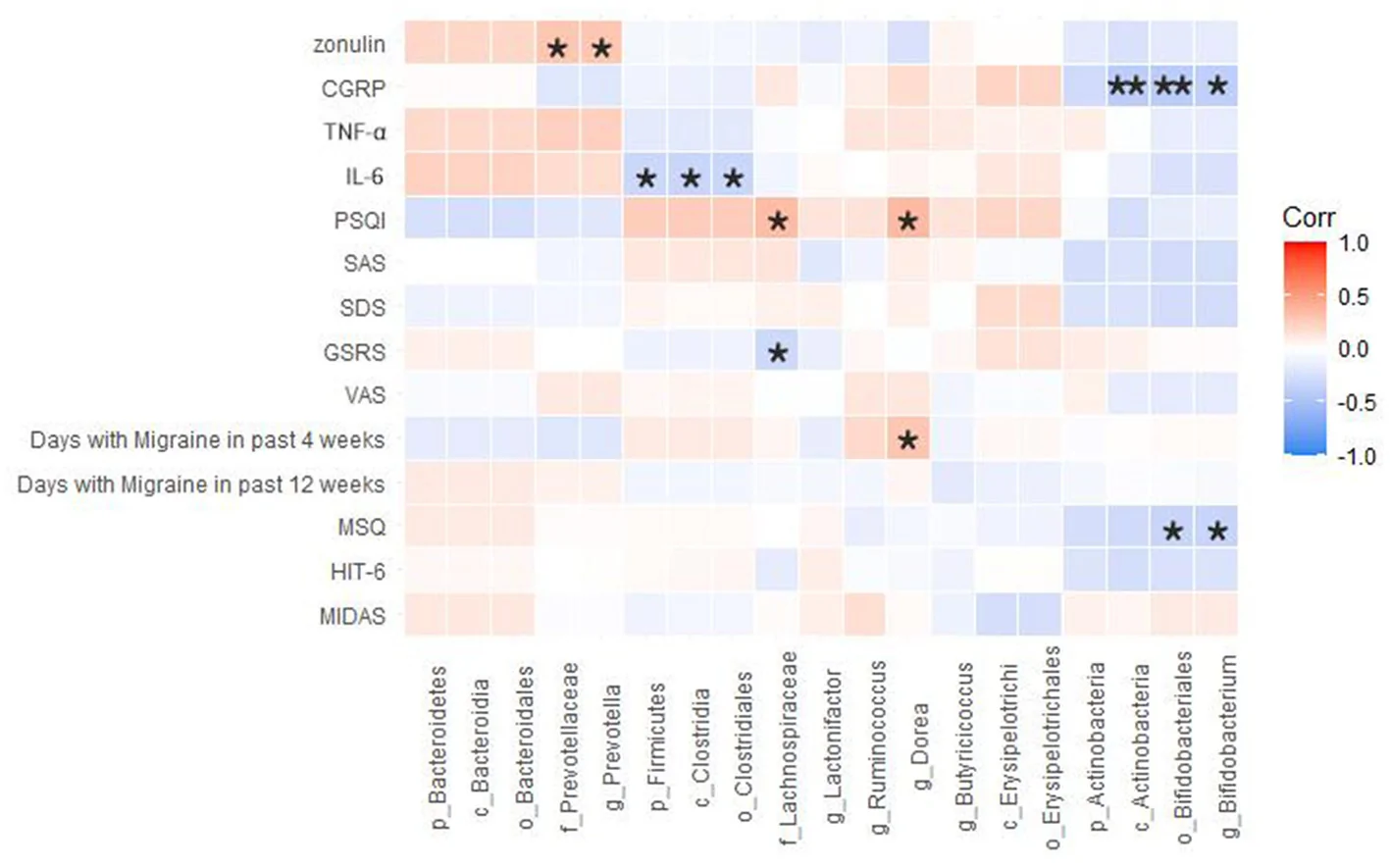
Correlations heatmap of gut microbiota. Heatmap shows correlations between gut microbiota and clinical characteristics, IL-6, TNF-α, CGRP and zonulin. The intensity of the color represents the r value (correlation coefficient; negative score: blue; positive score: red). Significant correlations are marked with *. *P < 0.05, **P < 0.01.

A correlation analysis on the relative abundance of gut microbiota and symptom scores of migraine and its comorbidities, as well as the serum biomarker concentrations (IL-6, TNF - α, CGRP, and zonulin) was conducted in the true diet group as exploratory findings. We compared the correlation between gut microbiota and other variables in terms of the difference between the endpoint and baseline. As shown in the heatmap (Figure 6), there were significant negative correlations between the relative abundance differences of Proteobacteria, Bifidobacteriales and Bifidobacterium and the concentration difference of CGRP (r = −0.4011, *P* = 0.0063; r = −0.3963, *P* = 0.007; r = −0.3789, *P* = 0.0103). Bifidobacteriales and Bifidobacterium were also significantly negatively correlated with the difference of MSQ (r = −0.3338, *P* = 0.0251; r = −0.3322, *P* = 0.0258). There was significant negative correlation trend between differences of Bifidobacteriales and Bifidobacterium and SAS, SDS and IL-6: (r = −0.2663, *P* = 0.0771; r = −0.2707, *P* = 0.072; r = −0.2314, *P* = 0.1262) and (r = −0.2696, *P* = 0.0733; r = −0.2587, *P* = 0.0861; r = −0.2324, *P* = 0.1245) in addition.The relative abundance difference of Firmicutes, Clostridia and Clostridiales was significantly negatively correlated with the difference of IL-6 concentration (r = −0.3111, *P* = 0.0375; r = −0.3235, *P* = 0.0302; r = −0.3241, *P* = 0.0299); The difference of relative abundance of Lachnospiraceae was significantly negatively correlated with the difference in GSRS (r = −0.2965, *P* = 0.048), while it was significantly positively correlated with PSQI (r=0.3523, *P* = 0.0176); The relative abundance difference of Dorea was significantly positively correlated with the difference of headache days in the past 4 weeks and PSQI(r = −0.3004, *P* = 0.045; r = 0.3646, *P* = 0.0138). The difference of the relative abundance of the Prevotellaceae and Prevotella was significantly positively correlated with the difference of zonulin concentration (r = 0.3024, *P* = 0.0435; r = 0.3018, *P* = 0.0439). What is more, the differences of relative abundance of the order, family, and genus belonging to the Bacteroidetes all had positive correlation trend with TNF - α, IL-6, and zonulin.

## 4 Discussion

First, the most notable impact of the two diets on changes in gut microbiota structure was the opposing changes in relative abundance of the two dominant phylum, Firmicutes and Bacteroidetes. The true diet increased Firmicutes while decreasing Bacteroidetes, whereas, the opposite pattern was observed in the sham diet. The relative abundance of Bacteroididia, Bacteroidales, Prevotellaceae and Prevotella decreased, while Erysipelotrichi and Ruminococcus increased in the true diet group; The relative abundance of Clostridia, Clostridiales and Ruminococcus decreased, while Fusobacteriia significantly increased in the sham diet group, which were the main reasons for the changes in the levels of the two phylum. The significant change in Simpson index after true diet may also be related to this.

Firmicutes and Bacteroidetes together constitute the vast majority of human gut bacteria, representing Gram positive and Gram negative populations, respectively (16). Firmicutes and Bacteroidetes are closely related to the inflammatory response of the body, forming mutually reinforcing symbiotic relationship that may jointly promotes the host's adverse response to sensitive foods and inflammatory state (11). Firmicutes have potential anti-inflammatory effects. Some species in Firmicutes can produce the butyrate, which is a short chain fatty acid that primarily plays an immunomodulatory role. The reduction of short chain fatty acids may be linked to intestinal barrier dysfunction. The weakening of this natural barrier function can lead to exposure to various antigenic substances, then the fragile intestine becomes a source of inflammation (17, 18). And because all species in Bacteroidetes are Gram negative, their outer membranes contain the toxin lipopolysaccharides, which are known for their pro-inflammatory properties (19). The imbalance between Firmicutes and Bacteroidetes may induce immune inflammatory responses, which may be related to the pathogenesis of migraine (9).

Similarly, multiple clinical studies have shown the levels of Firmicutes and Bacteroidetes are associated with mental and digestive disorders. The overall content of Firmicutes in patients with depression is significantly lower than that in the health group (11, 20). The most consistent change in generalized anxiety disorder and major depression is the depletion of Firmicutes (21). It was reported that that Bacteroidetes exhibit pro-inflammatory properties and affect cytokine production due to endotoxins, leading to irritable bowel syndrome (17).

Our study found that the relative abundance of Ruminococcus increased significantly in the true diet group and decreased significantly in the sham diet group after 12 weeks, which also promoted different trends in the increase/decrease of Firmicutes between the two groups. Vuralli et al. found that compared to the control group, the abundance of Ruminococcus was lower in chronic migraine patients with drug overuse headache. Ruminococcus has also been found to be negatively correlated with inflammatory markers, and the relative consumption of Ruminococcus is related to systemic inflammatory status (22, 23). Negative health outcomes are often associated with a decrease in Ruminococcus, including Crohn's disease (23), depression and anxiety (24). Based on this, we speculated that the improvement in migraine and its comorbidities observed in the true diet group in this study may be related to the increased abundance of Ruminococcus, which alleviated the inflammatory state.

It is worth emphasizing that after 12 weeks of true diet, we observed a significant increase in the relative abundance of probiotic Bifidobacterales, Bifidobacterium and Butyricicoccus. A Mendelian randomization analysis showed that Bifidobacteriales are associated with a reduced risk of migraine, migraine with aura and migraine without aura. Bifidobacterium dysbiosis has been suggested to have a potential association with the development of migraine and was linked to a reduced risk of migraine. Butyricicoccus may be a protective factor for migraine without aura (25). Several studies have confirmed that supplementing probiotics such as Bifidobacterium may alleviate symptoms of migraine (26, 27), reduced the occurrence of anxiety and depression symptoms (21), and the Bifidobacterium had a therapeutic effect on regulating the gut microbiota of patients with inflammatory bowel disease (17). These findings support the results of our study.

Butyricicoccus has been clinically proven to be a safe butyrate producing microbiota (15). Butyrate not only has beneficial effects on the intestine but also on the nervous system through the gut brain axis. For example, changes in bacteria that produce butyrate can regulate peripheral and central nervous systems as well as brain function (28). The beneficial effects of probiotics on clinical symptoms may be related to the promotion of increased epithelial integrity and decreased levels of pro-inflammatory cytokines, as probiotics have an impact on the production of short chain fatty acids (29). These research results, together with the findings of this study, suggest that Bifidobacterium and Butyricicoccus may play a potential role in the development of migraine and its comorbidities, and may be promising therapeutic targets.

Notably, we performed exploratory correlation analyses to examine potential links between changes in the relative abundance of intestinal bacteria and serum biomarker concentration, clinical symptom scores before and after true diet. We observed four statistically significant correlations:

- The increase of Actinobacteria, Bifidobacteriales and Bifidobacterium were associated with a decrease in MSQ and CGRP, and there was also a significant trend of co variation with a decrease in SAS, SDS, and IL-6. The gut microbiota has been suggested to play a potential role in the pathogenesis of migraine, depression, and anxiety (30). Clinical studies have found that the use of probiotic mixtures containing Bifidobacteriales can reshape the gut microbiota, reduce intestinal inflammation, improve barrier function, enhance mucosal integrity, significantly increase the relative abundance of bifidobacteria and improve symptoms of migraine, depression, anxiety, and sleep disorders (21, 26, 27, 31), which supports the results of our study. There is research confirming that gut microbiota regulates visceral sensitivity through the production of CGRP (32).
- The magnitude of the increase in Firmicutes, Clostridia and Clostridiales was positively correlated with the magnitude of the decrease of IL-6. While gut microbiota belonging to Bacteroidetes showed decrease in the same direction as IL-6. Based on our earlier observations, the increase in Firmicutes and the decrease in Bacteroidetes after true diet may be explained by reduced systemic inflammation linked to food specific IgG-mediated reactions, as well as changes in the concentrations of TNF - α and IL-6. The gut microbiota belonging to Firmicutes, which have potential anti-inflammatory effects, and Bacteroidetes, which can promote inflammation, may also be involved in this process. The reversal of their composition ratio after true diet may promote the process of systemic inflammation relief (12, 18, 33). Interestingly, Tang et al. found that chronic migraine like pain caused by dysbiosis of the gut microbiota is achieved through upregulation of TNF - α levels (34). However, our study found correlation between the relative abundance of gut microbiota and changes in pro-inflammatory cytokines after ture diet, with IL-6 being more significant than TNF - α. This may be related to the fact that over 90% of the participants in our study were patients with episodic migraine, and further clinical studies with richer subtypes are needed to confirm this.
- Zonulin showed decrease in the same direction with phylum, order, family, and genus belonging to Bacteroidetes, expecially with the significant correlation with Prevotellaceae and Prevotella. Serum zonulin is a biomarker for intestinal permeability deficiency (35). Studies have confirmed that food can trigger excessive production of zonulin (36), which leads to a process that tight junction barriers loosen and intestinal permeability increases, allowing intestinal bacteria and incompletely digested nutrients to enter the bloodstream from the intestinal lumen (37). The immune system may produce specific IgG antibodies against nutrients and induce food specific IgG-mediated reactions (38). This situation can lead to an increased immune response, thereby inducing the release of pro-inflammatory cytokines (39, 40). Accordingly, there is a bidirectional relationship between increased intestinal permeability and inflammation (41). The pro-inflammatory properties of Bacteroidetes may interact with zonulin, and both showed a decrease after true diet. Therefore, we speculate that Bacteroidetes may be potentially associated with food specific IgG-mediated reactions, as well as the reduction of intestinal wall permeability linked to the alleviation of systemic inflammation.
- The increase of Lachnospiraceae and Dorea showed a reverse change with the decrease in PSQI, and the same reverse change also occurred between Dorea and the number of migraine days in the past 4 weeks. A recent study found that Dorea was more abundant in chronic migraine patients than in healthy individuals. Dorea can metabolize sialic acid located at the end of mucins, and release of sialic acid is related to mucin degradation, thereby increasing intestinal permeability (22). There are also studies confirming that the abundance of Lachnospiraceae and Dorea increased in patients with sleep disorders (42, 43). These potential mechanisms may be related to the adverse effects of increased abundance of Lachnospiraceae and Dorea on sleep quality and improvement of headache duration.

The microbiota gut brain axis (MGBA) may provide a theoretical framework for current findings. The microbiota gut brain axis includes the brain, gut, immune cells, and gut microbiota, which communicate bidirectionally to maintain homeostasis (27, 44, 45). The ecological imbalance of gut microbiota may be associated with host derived cytokines and low-level inflammation throughout the body, which not only affects the intestinal epithelial barrier and digestive system, but cytokines may also have the potential to influence brainfunction, potentially contributing to pathogenesis of host neurological diseases such as pain, depression, anxiety, etc. (30, 46, 47). Our prior work speculate that IgG positive food elimination diet may lead to reduced antigen exposure, potentially reduced food specific IgG-mediated reactions, and reduced systemic inflammation (5). The reduction of pro-inflammatory factors may be associated with downregulation the release of zonulin and CGRP. The decrease in zonulin concentration was associated with reduced the permeability of the intestinal epithelial barrier, which may have contributed to improvement in the structure of gut microbiota - the Firmicutes increased while the Bacteroidetes decreased, and the relative abundance of some probiotics increased. Ultimately, there was an improvement in clinical symptoms of migraine, sleep, and gastrointestinal dysfunction. More importantly, considering that the crosstalk between the gut and the brain in the microbiota gut brain axis is bidirectional communication (6, 48), we propose that the elimination diet therapy mode of fasting IgG positive foods may form a positive feedback virtuous cycle, where multiple links involved in this process may interact with each other.

Several limitations of this trial should be acknowledged. First, although this model is an insightful hypothesis, it remains speculative in the context of our current study, and further research is needed to validate these proposed interactions and thorough mechanistic explanation such as specific signaling pathways. Second, intergroup baseline microbiota imbalances, combined with the possibility of regression toward the mean, may have contributed to some of the observed post-intervention differences (49, 50). These factors should be fully considered when interpreting the results. Third, given the limited sample size, multiple statistical tests performed, and moderate correlation coefficients (r ≈ 0.3–0.4), the associations identified herein are preliminary and hypothesis-generating rather than confirmatory. Further validation in larger, adequately powered studies using contemporary microbiome analytical approaches is therefore warranted. Notably, the absence of correction for multiple comparisons in microbiota and correlation analyses increases the risk of false-positive findings. Accordingly, the reported results should be interpreted with appropriate caution as exploratory observations. Independent validation in larger cohorts with appropriate multiplicity correction is necessary before definitive conclusions can be drawn. In addition, parametric tests relying on the central limit theorem (CLT) cannot eliminate biases from compositional non-normal microbiota data, which may be reduced in future via specialized statistics or large-sample dual parametric-nonparametric analysis.

## 5 Conclusions

In summary, we provide a clinical evidence that IgG positive food elimination diet altered gut microbiota, which was associated with decreased zonulin, CGRP and IL-6, and improvements in migraine and sleep. Our findings suggest the potential utility of gut microbiota analysis as a tool for generating preliminary mechanistic hypotheses, which are potentially linked to food-specific IgG-guided elimination strategy. Future work should identify more promising biomarkers and enlighten new pathways of elimination strategy for migraine based on the microbiota gut brain axis theory.

## Data Availability

The data presented in the study are deposited in the CNCB-NGDC Genome Sequence Archive (GSA) repository, accession number HRA019798 (51, 52).
