# Supplementary material for: IgG positive food elimination diet altered gut microbiota, decreased zonulin in migraine adults: a pilot dietary intervention trial
Source: Front Nutr. 2026 Aug 12;13:1878772. doi: 10.3389/fnut.2026.1878772 (PMC13506726; doi:10.3389/fnut.2026.1878772)
Supplement: Supplementary file 1 [file Supplementary_file_1.doc]

**Supplementary Table 1 . Baseline demographic and characteristics by two diet groups**

| **Characteristic [1]** |  | **Sham Diet Group (n = 39)** |  | **True Diet Group**  **(n = 45)** | **p-value [2]** |
| --- | --- | --- | --- | --- | --- |
| Age at enrollment |  | 40.8 (7.82) |  | 38.4 (9.19) | 0.1765 |
| Male n (%) |  | 12 (30.8) |  | 15 (33.3) | 0.7862 |
| BMI (kg/m^2^) |  | 24.1 (3.33) |  | 23.7 (3.8) | 0.3213 |
| With aura, n (%) |  | 3 (7.69) |  | 9 (20) | 0.0752 |
| Chronic migraine, n (%) |  | 4 (10.26) |  | 4 (8.88) | 0.8763 |
| Time since diagnosis at enrollment (month) |  | 92.6 (71.43) |  | 115.2(104.18) | 0.2551 |

[1]Continuous endpoints are summarized using mean (SD); Categorical endpoints are summarized using count (%).

[2] P value is from independent two group’s t test for continuous endpoint and chi square test for categorical endpoint.

**Supplementary Table 2. Differences of α- Diversity between Baseline and Week 12 in two Groups**

| α- Diversity | Sham diet T12  vs. Sham diet T0  (n = 39) | Ture diet T12  vs. Ture diet T0  (n = 45) |
| --- | --- | --- |
| chao1 | H=1.286520  P=0.256690 | H=0.222857  P=0.636871 |
| Faith_PD | H=2.445984  P=0.117826 | H=0.510037  P=0.475123 |
| observed_OTUs | H=1.230656  P=0.267279 | H=0.159580  P=0.689543 |
| Shannon | H=0.188975  P=0.663771 | H=2.216703  P=0.136524 |
| Simpson | H=0.171997  P=0.678343 | H=4.021311  P=0.044929 |

H：the calculation test statistic

**Supplementary Table 3. Differences of β- Diversity between Baseline and Week 12 in two Groups**

| β- Diversity | Sham diet T12  vs. Sham diet T0  (n = 39) | Ture diet T12  vs. Ture diet T0  (n = 45) |
| --- | --- | --- |
| Bray-Curtis Distance | F=1.282972  P=0.141 | F=1.581849  P=0.046 |
| Weighted UniFrac Distance | F =0.709747  P=0.561 | F =3.257259  P=0.020 |
| unweighted-unifrac-body Distance | F =0.877698  P=0.527 | F = 4.198111  P= 0.001 |

F: The sum of squared distances between samples from different groups divided by the sum of squared distances between samples from the same group
